# Supplementary material for: Statistical methods for predicting the presence of Salmonella Typhi in wastewater samples at Asante Akyem Agogo, Ghana
Source: PLoS Negl Trop Dis. 2026 Feb 17;20(2):e0013973. doi: 10.1371/journal.pntd.0013973 (PMC12923122; doi:10.1371/journal.pntd.0013973)
Supplement: Attachment — Submitted filename: Response to Reviewers_PNTD-D-25-01304.pdf [file pntd.0013973.s001.pdf]

Manuscript ID: [PNTD-D-25-01304]

Title: Statistical Methods for Predicting the Presence of *Salmonella Typhi* in Wastewater Samples at Asante Akyem Agogo, Ghana

Journal: PLOS Neglected Tropical Diseases

Corresponding Author: Sampson Twumasi-Ankrah

Dear Editor and Reviewers,

Thank you for the opportunity to revise our manuscript. We are grateful to the reviewers for their thoughtful and constructive comments, which have significantly improved the quality of our work. We have carefully addressed each point raised by the editor and Reviewer #1. Below, we provide a point-by-point response detailing the changes made.

Journal Requirements

1. Author Summary
   We have added an Author Summary section (approx. 150 words) between the Abstract and Introduction. This summary highlights the study's objectives, key findings, and public health implications in language accessible to both scientists and non-scientists.
   Location: Page 2, after the Abstract.
2. Figure Files
   All main figures (Fig 1, Fig 2, Fig 3) have been prepared as separate high-resolution .tif files in accordance with PLOS formatting guidelines. These will be uploaded separately during submission.
3. Financial Disclosure
   We have revised the Financial Disclosure statement as requested:

- Specified the grant number and recipient initials: "[INV-002381 to N.C.G.]."
- Clearly stated the funder's role: "The funders had no role in study design, data collection and analysis, decision to publish, or preparation of the manuscript." Location: Before the References section.

4. Competing Interests
   The Competing Interest statement has been updated to the standard PLOS format: "The authors have declared that no competing interests exist." Location: After the Financial Disclosure.

5. Table Numbering
   We have renumbered all tables sequentially to address the gap between Table 2 and Table 5.

    - Original Table 5 is now Table 3
    - Original Table 6 is now Table 4
    - Original Table 7 is now Table 5
      All in-text citations have been updated accordingly.

Reviewer #1 Comments

Comment 1: *Clarify methodological details such as variable selection criteria, model validation parameters, and data partitioning into training/testing subsets.* Response: We have expanded the Materials and Methods section to provide greater transparency.

- Variable selection criteria are now explicitly stated in the *Measurement of Outcome and Independent Variables* subsection (page 5).
- Model validation and data partitioning details have been added to the *Data Preparation* subsection (page 6–7), including a clear description of the stratified 5-fold cross-validation approach, site-level stratification, and hyperparameter tuning.
- The *Identifying Key Risk Factors Using SHAP Analysis* subsection (page 7–8) has been revised to align with the provided R code, specifying the use of lagged variables, the ranger package, and SHAP computation via fastshap.

Comment 2: *Expand the discussion of study limitations, including sample size, seasonality, and potential overfitting concerns given the high accuracy metrics (Accuracy > 0.99).* Response: We have expanded the Limitations and Future Research section (page 15) to explicitly address:

- Uneven sampling across towns and its effect on generalizability.
- The potential for overfitting due to high accuracy metrics, and the mitigation strategies employed (site-stratified cross-validation, hyperparameter tuning, out-of-bag error estimation).
- The limitation of the HF183 marker in distinguishing typhoid carriers.

Comment 3: *Slightly reinforce the interpretation of ecological and biological mechanisms underlying S. Typhi persistence in environmental contexts.* Response: In the Discussion section (page 14), we have strengthened the interpretation of how pH, dissolved oxygen, and seasonal rainfall influence *S. Typhi* survival and detection.

Comment 4: *In the Conclusion, emphasize the potential contribution of the model to practical public health systems (GHSA, WHO GLASS).*
Response: The Conclusion (page 15) has been revised to highlight the model's potential for integration into global health surveillance frameworks: "This model holds promise for practical implementation in public health surveillance networks, such as the Global Health Security Agenda (GHSA) and WHO GLASS, enabling proactive, environment-based typhoid monitoring in endemic regions."

Additional Minor Revisions

- We have ensured that all references to tables and figures are consistent and correctly numbered.
- Grammatical and typographical errors have been corrected throughout the manuscript.

Compliance with PLOS Guidelines

- All supporting data are publicly available via OSF (DOI: 10.17605/OSF.IO/WGPUM).
- Ethical approval is clearly stated in the *Ethics* subsection.
- The manuscript now includes the required Author Summary, Financial Disclosure, and Competing Interests statements in the prescribed format.

We believe that these revisions have thoroughly addressed all editorial and reviewer comments, enhancing the manuscript's clarity, reproducibility, and impact. Thank you again for your valuable feedback. We look forward to your consideration of our revised submission.

Sincerely,

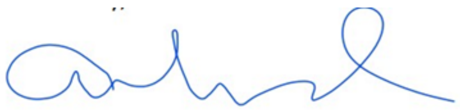

Sampson Twumasi-Ankrah (PhD)
On behalf of all authors
